# Does donating human milk result in greater compromise to the bone mass of breastfeeding mothers?

**DOI:** 10.1016/j.jped.2026.101604

**Published:** 2026-09-05

**Authors:** Keny Gonçalves Tirapeli, Larissa Brazolotto Ferreira, Carla Cristiane Silva, José Eduardo Corrente, Tamara Beres Lederer Goldberg

**Affiliations:** a Postgraduate Program in Tocogynecology, Faculdade de Medicina de Botucatu, Universidade Estadual Paulista (UNESP), Botucatu, SP, Brazil; b Department of Human Movement Studies, Universidade Estadual de Londrina (UEL), Londrina, PR, Brazil; c Department of Statistics, Instituto de Biociências, Universidade Estadual Paulista (UNESP), Botucatu, SP, Brazil; d Postgraduate Program in Tocogynecology, Department of Pediatrics, Faculdade de Medicina de Botucatu, Universidade Estadual Paulista (UNESP), Botucatu, SP, Brazil

**Keywords:** Alkaline phosphatase, Bone mineral density, Bone resorption, Human milk banks, Lactation, Osteocalcin

## Abstract

**Objective:**

Although the effects of lactation on bone mass are frequently discussed, it is unknown whether these effects are intensified in breastfeeding mothers (BFM) who become donors (BFM-donors). Thus, the objective was to evaluate alterations in bone densitometry and bone metabolism in BFM-donors during six months of follow-up, compared with BFM-controls who did not donate maternal milk, to determine whether these changes would be intensified by the volume donated.

**Methods:**

Data were obtained from two groups: 39 BFM-donors (six and twelve months postpartum) and 38 BFM-controls (up to 15 days [baseline] and six months postpartum). Donated milk volume, body mass index, and bone mineral density (BMD) of the lumbar spine, proximal femur, and total body were assessed by densitometry. Calcium, phosphorus, parathyroid hormone, 25(OH)D, estradiol, osteocalcin, bone alkaline phosphatase, and carboxy-terminal telopeptide were measured. Comparisons were performed using Student’s *t*-test, followed by repeated-measures models and ANCOVA adjusted for confounders when appropriate. Effect size and statistical power were also calculated.

**Results:**

BFM-donors showed bone mass mobilization similar to BFM-controls at six months, without influence of donated volume (9.0 ± 8.2 L). From six to 12 months postpartum, lumbar spine and femur BMD values increased (5% and 1.5%).

**Conclusions:**

Comparing BFM-donors at 12 months, versus BMF-controls at 15 days, even with continued complementary breastfeeding, densitometric results tended to return to the means seen 15 days postpartum. There were no correlations between BMD, bone markers, and total volume of donated milk.

## Introduction

Breastfeeding (BF) is a natural bonding and nutrition strategy, considered the most effective intervention for reducing infant morbidity and mortality. Exclusive breastfeeding (EBF), is recommended until the child is six months old, and supplemented breastfeeding (SBF), when, in addition to human milk (HM), the child receives other foods, should be continued until the age of two years of life or more [1].

To meet the child's calcium demands, milk production can take place through mobilization of the maternal skeleton. For this reason, lactation is recognized as one of the main stimuli for increasing bone mobilization, an effect that is most evident in trabecular bones [2].

When breastfeeding is carried out exclusively, reductions of between 3% and 10% are observed in BMD of the lumbar spine [3,4]. After switching from EBF to SBF, six months postpartum, there is a tendency to bone recovery, [[5], [6], [7]] however, the speed at which this occurs depends on the affected skeleton site, type of BF employed, [8] and, assumedly, amount of milk produced [9].

Human Milk Banks (HMBs) are specialized, non-profit institutions responsible for recruiting donors, collecting and rigorously processing (Pasteurization) all donated HM, [10] to control the quality, and distributing the HM. HMBs also work to promote and protect breastfeeding, being considered an important public health strategy in reducing child morbidity and mortality [11,12]. Brazil is highlighted internationally as a reference for the Global Network of HMBs.

To our knowledge, this is the first study to investigate bone metabolism and bone mineral density in HM donors. Despite the public health importance of HM donation, its effects on maternal bone health remain unknown. Specifically, it is unclear whether the additional physiological demands of frequent milk expression, combined with exclusive breastfeeding of their own infants, result in greater bone mineral mobilization than in mothers who exclusively breastfeed without donating milk.

Thus, the objective of the current study was to evaluate whether HM donation is related to additional changes in bone mineral density and bone remodeling markers during lactation.

## Methods

This study is a subproject of a larger investigation, that prospectively and longitudinally monitored the subgroup of BFM donors from an HMB using a descriptive and analytical approach, approved by the University’s Research Ethics Committee (CAAE: 20372619.3.0000.5411).

Considering the study by Costa et al., [13] for a standard deviation (SD) of the distal radius of 0.0057 g/cm^2^, alpha of 5%, power of 90%, and difference between measurements of 0.010 g/cm^2^, a minimum sample size of 36 participants per group was defined.

**Figure 1:**
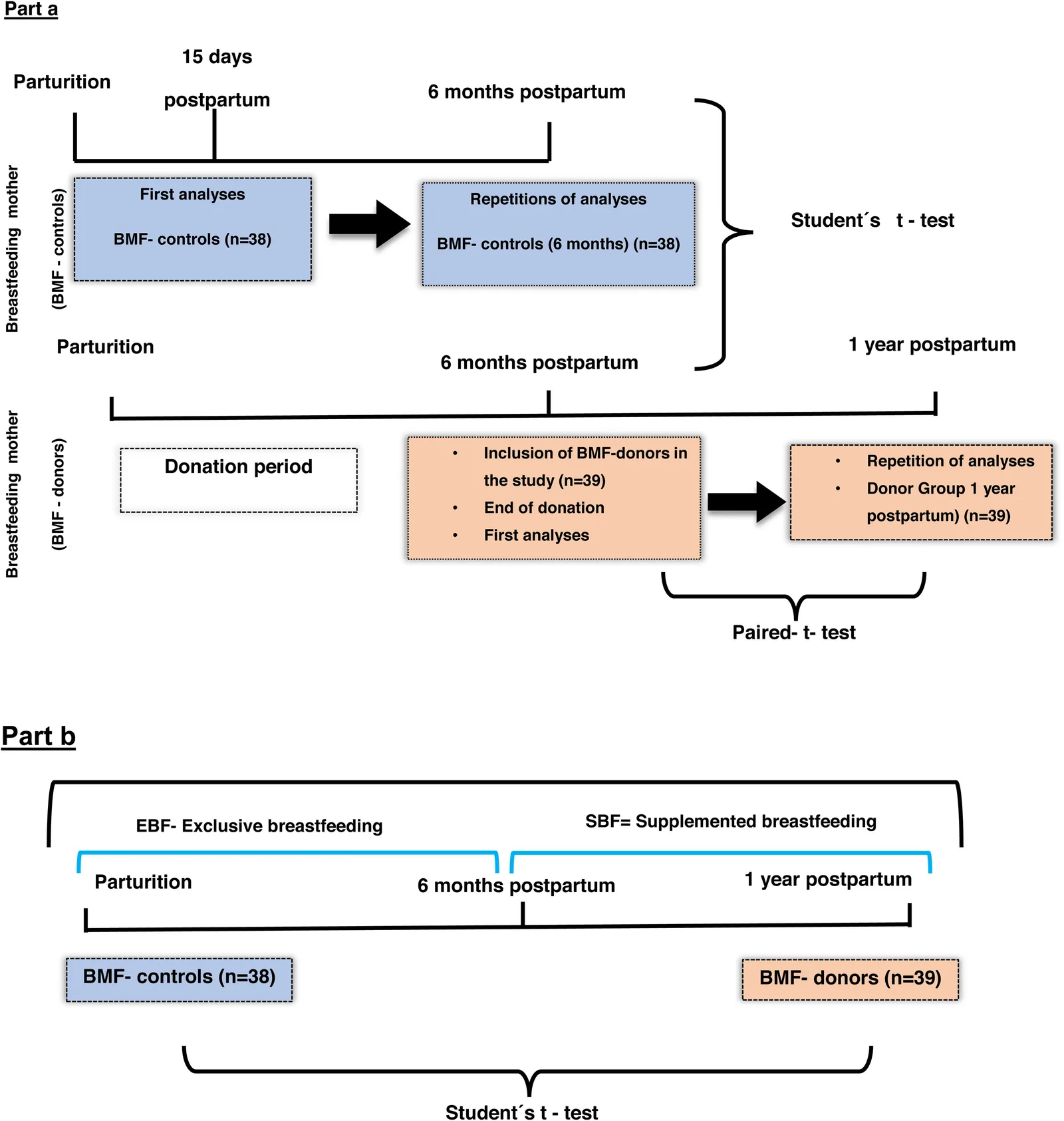
Flowchart of study groups and monitoring models (Part a and b). BFM-controls, BFM-donors, EBF, Exclusive breastfeeding; SBF, Supplemented breastfeeding.

Data collection took place from July 2020 to March 2023. Participants who voluntarily signed the informed consent form were allocated into two groups: BFM-controls (n = 38), exclusively breastfeeding mothers evaluated within 15 days postpartum, and again at six months postpartum; and BFM-donors (n = 39), mothers who donated HM from shortly after delivery until six months postpartum, while maintaining exclusive breastfeeding. BFM-donors were required to intend to continue complementary breastfeeding until at least one year postpartum. The same BFM-donors were re-evaluated at 12 months postpartum after discontinuing milk donation and continuing complementary breastfeeding. To evaluate bone mass changes over time, comparisons were performed between BFM-controls and BFM-donors at six months postpartum; between BFM-donors at six and 12 months postpartum; and between BFM-controls at baseline and BFM-donors at 12 months postpartum (Figure 1).

To avoid undesirable effects on bone mass resulting from previous pregnancies, only primiparous women were included, with usual risk prenatal care, who had a full-term newborn, with appropriate weight for gestational age. The self-reported pre-pregnancy Body Mass Index (BMI) was eutrophic or overweight and the age range was 18 to 38 years, to minimize effects on bone mass resulting from advancing age.

The non-inclusion criteria were the presence of diseases that could compromise bone metabolism, and the use of medications or calcium and/or vitamin D supplements (in the years studied, breastfeeding mothers monitored within the Unified Health System (SUS) did not receive prescriptions for Calcium and Vitamin D, as these were not recommended in the public service guidelines in Brazil), which could also affect bone mass. Participants who did not attend the follow-up sessions or who, despite their previous intention to breastfeed, interrupted it early, were excluded.

At the first meeting, the participants answered a questionnaire containing sociodemographic information (family income, education level, pre-pregnancy BMI, frequency of sun exposure, age at menarche), and at all subsequent meetings, they were asked about the number of breastfeeds. For BFM-donors, the total volume of donated milk (in liters) and the donation period (in days) were recorded; obtained from HMB records.

The participants answered a validated questionnaire on their consumption frequency of foods that are sources of calcium and vitamin D (FFQ), and the size of the portions consumed [14]. Their responses were used to estimate the daily consumption of these nutrients, with the help of Dietwin Nutrition Software, comparing the results to the Dietary Reference Intakes/Recommended Dietary Allowance (DRIs/RDA) values for breastfeeding women; 1000 mg/day for calcium and 600 International Units (IU) for vitamin D [15].

Weight (kg) and height (meters) were measured at all meetings, to classify pre-pregnancy (participant report) and current nutritional status using the BMI (kg/m²): underweight: less than 18.5 kg/m²; eutrophic or normal: 18.5 to 24.9 kg/m²; and overweight: between 25 and 29.9 kg/m² [16].

Dual-energy x-ray absorptiometry (DXA), using a GE-Lunar DPX NT device, was used to measure the BMD (g/cm²) of the lumbar spine (L1-L4), total proximal femur, and total body, as well as body fat mass (kg), body fat percentage (%BF), and lean mass (LM) (in kg). The measurements were carried out by the same technician, who was blinded and had no knowledge of which group the participants belonged to, following the manufacturer's instructions and International Society of Densitometry standards [17]. The coefficient of variation (CV) was 0.86% for lumbar spine, 1.39% for femoral neck, and 1.00% for total body.

Blood was collected after fasting for 8 to 10 hours, between 7:00 and 9:00 in the morning. All measurements were carried out in serum, except Carboxy-terminal telopeptide (S-CTx) which was performed in ethylenediaminetetraacetic acid (EDTA) plasma.

BAP was measured by automated chemiluminescence (LIAISON® XL, DiaSorin; LIAISON® BAP OSTASE; CV 5.8%; reference 4.9–26.6 mcg/L), OC by electrochemiluminescence (Cobas E 801, Roche; Elecsys N-MID OC; inter-assay CV 2.7–5.1%, intra-assay CV 1.3%; reference 11–46 ng/mL), and S-CTx using an electrochemiluminescence assay (Cobas E 801, Roche; Elecsys β-CrossLaps; CV 1.1%; reference 0.025–0.573 ng/mL).

Total calcium and phosphorus were measured by automated photocolorimetry (LAB MAX/COBAS®; CV 0.68% and 2.18%; references 8.5–10.5 and 2.5–4.8 mg%, respectively). Hormones were analyzed by chemiluminescence (Atellica™ IM): PTH (CV ≤ 8%; 18.5–88.0 pg/mL), 25(OH)D (CV < 10%; per Endocrine Society recommendations), [18] and estradiol (CV ≤ 7%; follicular 93–575, luteal 43–214 pg/mL).

### Data analysis

Normality was assessed using the Shapiro–Wilk test (SAS for Windows, v.9.4). The sample is described using descriptive statistics, including frequency distributions for categorical variables and mean ± standard deviation (SD) for continuous variables. Sociodemographic and anthropometric variables were analyzed using the chi-square test and/or tests for differences in proportions.

The Student’s t-test was used to compare means between BFM-controls (Baseline) and BFM-donors (12 months postpartum). Paired t-tests were applied to compare BFM-donors at six and 12 months postpartum. The Student’s t-test compared BFM-controls and BFM-donors at six months for anthropometric (weight, height, BMI), hormonal, biochemical, densitometric, and bone marker variables. Pearson’s correlation was used to assess associations between total donated milk volume (L) and changes in bone mass and markers, as well as between calcium - and vitamin D-rich food intake and these outcomes.

Repeated-measures analysis of variance models were fitted to analyze the BFM-donor variables at six and 12 months postpartum, adjusted for potential confounding variables. Comparisons between BFM-controls and BFM-donors (12 months postpartum) were performed using analysis of variance and covariance (ANCOVA) models, adjusted for potential confounders. Finally, for variables showing statistically significant differences, effect size and statistical power were calculated.

## Results

**Figure 2:**
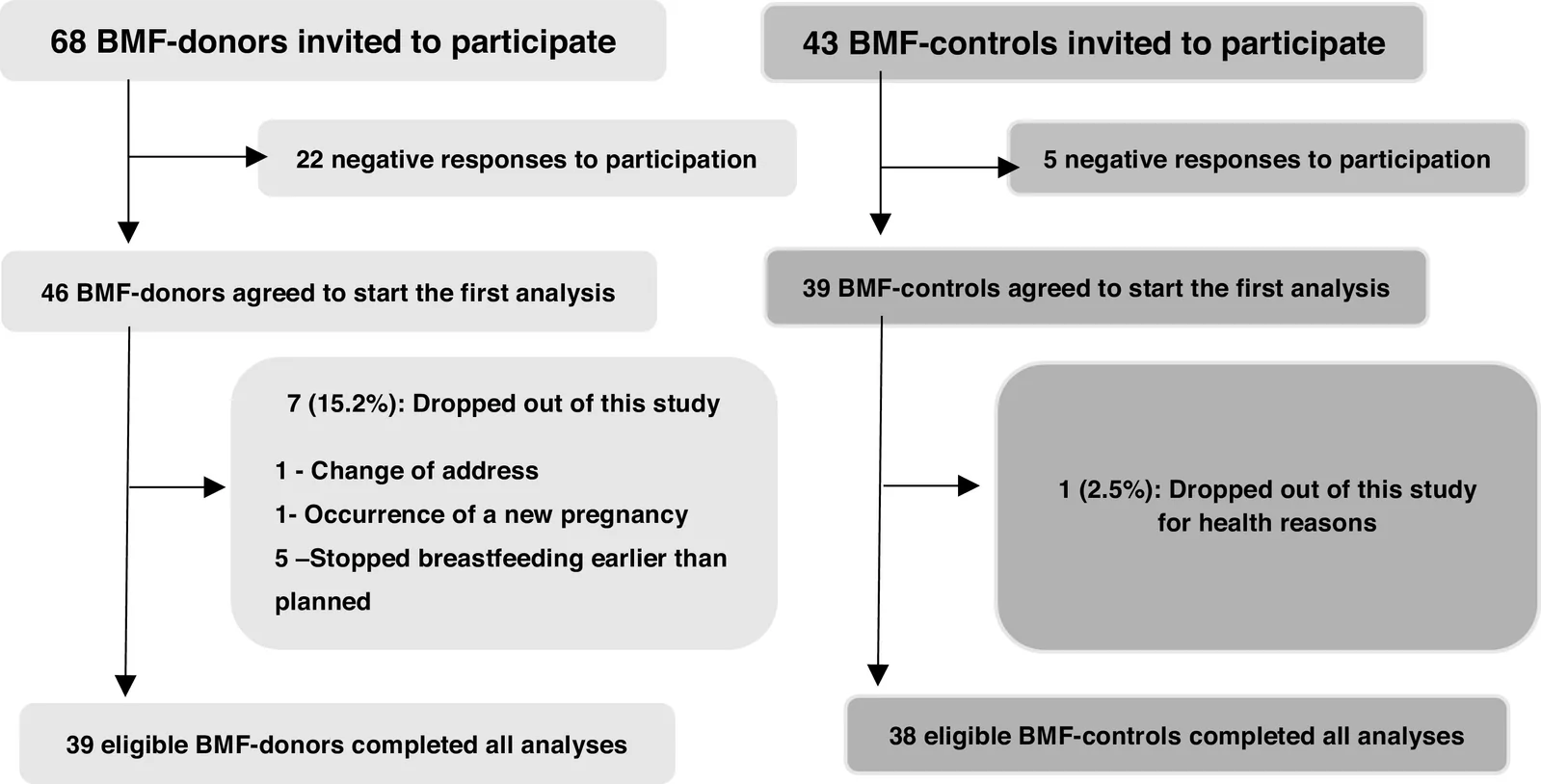
Participant recruitment flowchart.

Of the 68 BFM-donors invited to participate in the study, 46 were included in the first analyses and, of these, 15.2% (n = 7) did not continue participation. The reasons for discontinuity are presented in Figure 2.

Regarding the sociodemographic aspects of BFM-donors, 56.4% attended higher education, 74.4% worked, 30.7% had an income between 3 and 6 minimum wages (MW), and 97.4% were married/in a stable union. The majority declared their ethnicity/color as white (69.2%) and 71.7% underwent cesarean section, with no significant differences characteristics between BFM-controls and BFM-donors at the beginning of the study, except for education and marital status (Table 1).

**Table 1:** Sociodemographic, reproductive, lactating, and donation characteristics of BFM-donors and BFM-controls.

Variables	BFM- donors (n=39)		BFM-controls (n=38)		p-value
	15 days postpartum		15 days postpartum		
	N	%	n	%	
Education level					
Primary	4	10.2	1	2.5	0.300
Secondary	13	33.3	25	65.3	0.010
Higher	22	56.4	12	32.2	0.060
Employment occupation					
Yes	29	74.4	28	73.7	0.900
No	10	25.6	10	26.3	
Marital status					
Married/Stable Union	38	97.4	25	67.5	0.020
Single	1	2.5	13	34.2	0.000
Family income (MW)					
0	2	5.1	2	5.5	1.000
Up to 1	6	15.3	3	7.8	0.531
1 to 3	14	36.2	17	44.7	0.510
3 to 6	12	30.7	14	36.8	0.683
≥ 6	5	12.7	2	5.2	0.226
Ethnicity					
White	27	69.2	27	71.0	1.000
Mixed-race	8	20.5	10	26.3	0.737
Black	4	10.3	1	2.67	0.248
Type of birth					
Vaginal	11	28.3	16	42.1	0.163
Cesarean	28	71.7	22	57.9	
	Mean	SD	Mean	SD	
Age (years)	28.2	5.4	26.5	5.1	0.211^#^
Sun exposure (times/week)	4.0	2.0	4.6	2.5	

Variables: lactation, donation and anthropometry (Mean ± SD)					

	BFM-donors (6 months)	BFM-donors (one year postpartum)	BFM-controls (15 days postpartum)		

Number of breastfeeds per day	11.0 ± 3.3	6.1 ± 3.3	13.3 ± 2.0		
Volume of total donated milk (liters)	9.4 ± 8.2				
Total donation time (days)	116.4 ± 34.2				

Anthropometry					

Weight (kg)	65.9 ± 12.8	65.2 ± 12.8	68.1 ± 11.8	0.644^#^ 0.421& 0.825*	
Height (m)	1.62 ± 0.5	__________	1.61 ± 0.1	0.435^#^	
BMI (kg/m^2^)	25.1 ± 4.5	24.7 ± 4.7	26.2 ± 4.0	0.138^#^ 0.207& 0.867*	

***Note:*** Values presented as relative frequency (%). Association analysis x^2^ and test of difference in proportions for comparison between variables with more than two categories. ^#^ Student’s t-test, comparison between BFM-controls and BFM-donors

& Paired t-test, comparison between 6-month donors and one year donors; *ANOVA: Comparison between BFM-controls (up to 15 days postpartum), BFM-donors six months postpartum, and BFM-donors one year postpartum. SD, Standard Deviation; kg, kilograms; m, meters. Statistical significance p ≤ 0.05.

BFM-donors and BFM-controls did not show significant differences in age (p = 0.211), weight (p = 0.644), height (p = 0.435), and BMI (p = 0.138). When comparing BFM-donors six months postpartum to BFM-donors one year postpartum, there were no changes in weight (p = 0.421) or BMI (p = 0.207). Comparisons between BFM-controls, BFM-donors six months postpartum, and BFM-donors one year postpartum showed no differences in weight (p = 0.825) and BMI (p = 0.867) at any moment evaluated. The average daily feedings were 13.3 ± 2.0 times in the BFM-controls group, 11 ± 3.3 times six months postpartum (BFM-donors), and 6.1 ± 3.3 times one year postpartum (BFM-donors). An average of 9.0 ± 8.2 liters of milk were donated in 116.4 ± 34.2 days of registration by BFM-donors, with a median (IQR) of 5.8 (3.44–15.35) liters (Table 1).

No correlations were found between the total volume of milk donated with BMD from any site analyzed or with any of the bone biomarkers assessed (Supplementary Table 1).

The FFQ analysis showed no significant differences in daily calcium and vitamin D intake between BFM-controls and BFM-donors (p = 0.389; p = 0.268). Both groups had low intake (controls: 402.59±216.27 mg/day calcium; 102.1±70.02 IU/day vitamin D; donors: 455.08±320.93 mg/day calcium; 121.46±90.31 IU/day vitamin D), with no correlations with BMD at the evaluated sites.

### Comparisons of BFM-controls versus BFM-donors, both at 6 months postpartum

The EBF comparisons revealed no significant differences in BMD of the lumbar spine (L1-L4), total proximal femur, and total body, or in biochemical parameters of bone metabolism, except for phosphorus (adjusted p-value < 0.001), and 25(OH)D, both with statistical differences (adjusted p-value = 0.0347), with small and medium effect magnitudes, respectively. The body composition analysis also showed no significant differences in LM, FM, and body fat percentage (Table 2).

**Table 2:** Cross-sectional BMD and other data between BFM-donors and BFM- controls at 6 months postpartum.

Variables	BFM-donors 6 months postpartum	BFM-controls 6 months postpartum	p-value	Power (P) Effect size (ES)
	Mean ± SD	Mean ± SD		
Lumbar BMD (g/cm^2^) (L1-L4)	1.083 ± 0.121	1.075 ± 0.112	0.760	
Z score	-0.728 ± 1.073	-0.827 ± 1.033	0.724	
Total body BMD (g/cm^2^)	1.135 ± 0.090	1.119 ± 0.085	0.430	
Z score	0.235 ± 1.118	0.190 ± 1.009	0.865	
Femur BMD (g/cm^2^)	0.925 ± 0.129	0.929 ± 0.097	0.880	
Z score	-0.547 ± 1.056	-0.464 ± 0.831	0.737	
Ca (mg%)	8.85 ± 0.84	9.18 ± 0.55	0.054	
Phosphorus (mg%)	6.39 ± 4.72	4.12 ± 0.74	0.006	p<0.0001 adjusted P=0.8251 ES=0.5
PTH (pg/mL)	34.44 ± 18.29	37.01 ± 15.92	0.528	
25(OH)D (ng/mL)	23.75 ± 6.33	27.36 ± 8.96	0.047	p=0.0347 adjusted P=0.5264 ES=0.4
Estradiol (pg/mL)	56.53 ± 52.98	41.86 ± 46.91	0.367	
BAP (mcg/L)	20.79 ± 7.77	21.41 ± 10.62	0.772	
OC (ng/mL)	33.9 ± 10.53	36.86 ± 10.44	0.229	
S-CTx (ng/mL)	1.12 ± 0.41	0.98 ± 0.48	0.167	
LM (kg)	35.06 ± 4.09	35.33 ± 3.94	0.773	
FM (kg)	27.22 ± 9.52	25.06 ± 8.95	0.312	
% BF	42.57 ± 6.82	40.35 ± 8.07	0.198	

***Note:*** Student’s t-test, statistical significance p ≤ 0.05 (indicated in bold). SD, Standard Deviation; kg, kilograms; BMD, Bone Mineral Density; PTH, Parathormone; BAP, Bone Alkaline Phosphatase; OC, Osteocalcin; S-CTx, Carboxy-terminal telopeptide; LM, lean mass; FM, fat mass; %BF, percentage body fat. Adjustment variables: marital status and educational level, after adjustment using an analysis of covariance (ANCOVA) model.

### Comparisons of BFM-donors six months postpartum versus BFM-donors one year postpartum

Significant differences were observed in lumbar spine and total proximal femur BMD between the two moments evaluated, with higher values after 12 months (Table 3); 5.0 ± 3.9% (p < 0.001) in the lumbar spine and 1.5 ± 3.4% in the total proximal femur (p < 0.001). There were no significant differences in total body BMD.

**Table 3:** Comparison of densitometric, biochemical, and hormonal variables, bone markers, and body composition of BFM-donors 6 months versus BFM-donors one year.

Variables	BFM-donors 6 months postpartum	BFM-donors 12 months postpartum	p-value	Power (P) Effect size (ES)
	Mean ± SD	Mean ± SD	Paired t-test	
Lumbar BMD (g/cm^2^) (L1-L4)	1.083 ± 0.121	1.138 ± 0.111	<.001	p<0.001 adjusted P=1.0 ES= 0.45
Z score	-0.728 ± 1.073	-0.289 ± 0.917	<.001	p<0.001 adjusted P=1.0 ES= 0.41
Total body BMD (g/cm^2^)	1.135 ± 0.090	1.137 ± 0.084	0.348	
Z score	0.235 ± 1.118	0.183 ± 1.04	0.773	
Femur BMD (g/cm^2^)	0.925 ± 0.129	0.945 ± 0.129	0.001	p=0.004 adjusted P=1.0 ES= 0.15
Z score	-0.547 ± 1.056	-0.459 ± 1.061	0.046	p=0.00616 adjusted P=1.0 ES= 0.08
Calcium (mg%)	8.84 ± 0.84	9.19 ± 0.58	0.037	p=0.0439 adjusted P=0.49 ES= 0.42
Phosphorous (mg%)	6.39 ± 4.72	3.88 ± 0.57	0.001	p=0.0033 adjusted P=1.0 ES= 0.5
PTH (pg/mL)	34.44 ± 18.29	45.18 ± 21.11	0.001	p=0.0026 adjusted P=0.05 ES= 0.89
25(OH) D (ng/mL)	23.75 ±6.33	24.99 ± 10.81	0.515	
Estradiol (pg/mL)	56.53 ± 52.97	104.12 ± 99.98	0.055	
BAP (mcg/L)	20.78 ± 7.77	16.62 ± 7.052	0.001	p=0.0014 adjusted P=0.96 ES= 0.53
OC (ng/mL)	33.89 ± 10.52	30.70 ± 11.846	0.090	
S-CTx (ng/mL)	1.121 ± 0.40	0.631 ± 0.331	<.001	p<0.001 adjusted P=1.0 ES= 1.25
LM (kg)	35.06 ± 4.09	35.55 ± 3.54	0.011	p=0.0197 adjusted P=0.98 ES= 0.12
FM (kg)	27.22 ± 9.52	25.53 ± 9.87	0.017	p=0.0274 adjusted P=0.9 ES= 0.17
% BF	42.56 ± 6.81	40.40 ± 9.16	0.007	p=0.0136 adjusted P=1.0 ES= 0.23

***Note*:** Paired t-test (BFM-donors comparison 6 × 12 months postpartum); statistical significance p ≤ 0.05 (indicated in bold). SD, Standard Deviation; Kg, kilograms; BMD, Bone Mineral Density; PTH, Parathormone; BAP, Bone Alkaline Phosphatase; OC, Osteocalcin; S-CTx, Carboxy-terminal telopeptide; LM, lean mass; FM, fat mass; %BF, percentage body fat. Adjustment variables: marital status and educational level. Analyses were adjusted using a repeated-measures model.

Total calcium, phosphorus, and PTH levels differed significantly between the two time points. S-CTx showed a significant decrease after one year compared to six months, (adjusted p value ≤ 0.001, ES = 1.25). Significant changes were also observed in lean mass, fat mass, and body fat percentage (Table 3).

Significant increases were found of 3.9% in total calcium and 31.3% in PTH, and reductions in phosphorus, BAP, and S-CTx concentrations of 39.2%, 20.0%, and 43.7%, respectively, without significant differences in the concentrations of 25(OH)D, estradiol, and OC when comparing the moments.

No significant correlations were detected, according to the moments, between the concentrations of the biochemical markers (S-CTx, BAP, and OC) and BMD of each site evaluated (Supplementary Table 2).

### Comparisons of BFM-controls versus BFM-donors one year postpartum

Comparisons of lumbar spine BMD between BMF-controls at 15 days postpartum and BMF-donors at 12 months postpartum revealed no significant differences (adjusted p = 0.972, P = 1.0, and ES = 0.28) (Table 4).

**Table 4:** Comparisons between densitometric, biochemical, and hormonal variables and bone remodeling markers at the moments of analysis, exclusive breastfeeding mothers at 15 days postpartum (BFM-controls), and breastfeeding mother donors at 12 months postpartum (BFM-donors).

Variables	BMF-controls (Basal) (n=38)	BMF-donor 12 months postpartum (n=39)	p-value	Power (P) Effect size (ES)
	Mean ± SD	Mean ± SD		
Lumbar Spine BMD (L1-L4) (g/cm^2^)	1.107 ± 0.109	1.138 ± 0.111	0.048	p=0.9720 adjusted P=1.0 ES=0.28
Z-score	-0.621 ± 0.924	-0.289 ± 0.917	0.079	
Total Body BMD (g/cm^2^)	1.135 ± 0.086	1.137 ± 0.084	0.596	
Z-score	0.233 ± 0.997	0.183 ± 1.040	0.972	
Femur BMD (g/cm2)	0.961 ± 0.105	0.945 ± 0.129	0.441	
Z-score	-0.329 ± 0.844	-0.459 ± 1.061	0.778	
Ca (mg/dL)	9.09 ± 0.48	9.20 ± 0.59	0.675	
P (mg/dL)	4.31 ± 0.70	3.88 ± 0.57	0.024	
PTH (pg/mL)	24.64 ± 12.59	45.18 ± 21.12	<0.001	p<0.001 adjusted P=0.99 ES=0.97
25(OH)D (ng/mL)	30.01 ± 11.68	24.99 ± 10.82	0.123	
Estradiol (pg/mL)	51.33 ± 55.18	104.13 ± 99.98	0.007	p=0.0816 adjusted P= 0.80 ES= 0.53
BAP (mcg/L)	20.27 ± 11.76	16.62 ± 7.05	0.101	
OC (ng/mL)	32.18 ± 11.59	30.70 ± 11.85	0.056	
S-CTX (ng/mL)	1.02 ± 0.22	0.63± 0.33	<0.001	p<0.001 adjusted P=1.0 ES= 1.18

***Note:*** Student’s t-test. SD, Standard Deviation; g, Grams; BMD, Bone Mineral Density; BMC, Bone Mineral Content; PTH, Parathyroid Hormone; BAP, Bone Alkaline Phosphatase; OC, Osteocalcin; S-CTX, Carboxy-terminal telopeptide. Adjustment variables: marital status and educational level, after adjustment using an analysis of covariance (ANCOVA) model.

Regarding the biochemical analyses, the mean phosphorus (P) level at 12 months was 3.88 ± 0.57 mg/dL, compared with 4.31 ± 0.70 mg/dL in the BFM-controls. For PTH, the mean values differed significantly between the two moments (Table 4).

Estradiol and bone resorption S-CTx concentrations showed statistical differences between the two moments.

## Discussion

This is the first investigation into alterations in mineral density and bone metabolism in BFM-donors who exclusively breastfed their children for six months, and continued to breastfeed them in a complementary way for at least one year postpartum, with comparisons of the alterations between BFM-controls and BFM-donors. HM donation did not exacerbate lactation-related reductions in BMD.

The results of comparisons between BFM-donors at six months and at one year postpartum demonstrated near-complete recovery of BMD of the lumbar spine and total proximal femur, without changes in the total body BMD. Bone metabolism markers BAP and S-CTx showed significant reductions, without alterations in OC. After one year of BF, bone mass and markers tended toward recovery, except for PTH, estradiol, and S-CTx, low dietary intake of calcium and vitamin D were also found.

It would be reasonable to assume that the reduction in bone mass observed in BFM would be intensified in women who donated HM, in excess of that produced for their children. However, this hypothesis was not confirmed by the current study; differences were not observed in relation to BMD, bone markers, weight, BMI, or body composition.

Physiological mobilization in bone mass is observed during the gestational period and after birth, when it continues to advance, more intensely, as an effect of EBF [6]. In six months of EBF, alterations are described mainly in trabecular bones. In Brazil, only three studies evaluated BFM bone mass, [8,13,19] all indicating a tendency towards recovery of bone mass. In the sixth month postpartum, the switch from EBF to SBF explains a large part of the tendency towards recovery of bone mass verified among Brazilian donors and evidenced in the literature in BFM followed from the immediate postpartum until 12 months, with 1.21%;[19] 3.0%;[13] and 4.5% in BMD in the lumbar spine; 3.0% and 4.0% in BMD of the femoral neck [13,20]. Between six months and one year postpartum, increases of 4.0 to 5.0% in BMD of the lumbar spine were described, with no differences in total body BMD;[6] increases close to those found in BFM-donors from the present study.

Differences across studies likely reflect methodological heterogeneity, including the sites assessed, techniques, and, particularly, breastfeeding type and duration. Inconsistent group definitions — such as mixing exclusive and non-exclusive breastfeeding or varying weaning times — may also confound and obscure results [2,3,9,20]. However, findings in BFM indicate that bone mass recovery occurs regardless of weaning [6].

All these evolutionary trends are similar to those evidenced in the current study, despite the evaluation of different ethnicities and sites compared to other studies [3]. The percentage reductions in bone mass during exclusive breastfeeding, evidenced monthly during lactation, although transitory, are more significant than those observed in postmenopausal women, of 1 to 3% per year [4,5,7].

In the current study, BFM-donors provided an average of 9 liters of HM, quantities higher than those indicated in the Brazilian scientific literature, which reports from 0.9 to 1.4 liters per donor [21,22]. This difference may have arisen because the study was developed during the COVID-19 pandemic, which possibly contributed positively to donations, as social isolation provided greater convenience for the practice of BF. Despite the higher milk volume, no additional bone mass reduction beyond that expected from lactation itself was observed.

Soon after birth, alterations in bone marker levels are observed in breastfeeding mothers, earlier and more intensely in markers related to resorption and later, with smaller temporal variations, in those related to formation, such as FAO and OC, produced at different stages of osteoblast maturation. Acute phenomena can be better evaluated by OC concentrations, as BAP is more stable, requiring a longer period of monitoring, [23] as shown in the results found in BFM-donors, who presented an increase in this marker only during follow-up from six to 12 months postpartum.

BFM-donors at 12 months postpartum showed a significant difference compared to BFM-controls, indicating a reduction in bone resorption, evidenced by the beginning of SBF, when the child’s calcium needs are no longer exclusively supplied by breast milk.

With respect to hormonal alterations, it is known that the measurement of PTH, associated with serum calcium and 25(OH)D, allows greater precision in the diagnosis of bone changes. The progressive increases in levels of PTH, found at all moments evaluated in this study could have occurred in an attempt to maintain adequate serum calcium concentration [7].

The low dietary intake of calcium and vitamin D may have been the crucial factor for the detected increase in PTH, [24,25] which could reduce the absorption of calcium and phosphorus in the intestine, culminating in elevated PTH secretion. Adequate dietary intake of calcium by BFM provides optimal conditions for bone remodeling [26]. Cooke-Hubley et al. identified higher consumption (21.6 mcg, equivalent to 864 IU for vitamin D and 1,299.4 mg/day for calcium) compared to values obtained from BFM-donors (121.46 IU of vitamin D and 455.08 mg/day of calcium) [5,27].

Increased levels of prolactin during lactation suppress the hypothalamic-pituitary-gonadal axis, resulting in low levels of estrogen, one of the main stimulators of osteoclastic activity in BFM. As breastfeeding progresses, these values tend to normalize. An increase in estradiol levels was also observed one year postpartum in BFM-donors, reflecting an expected increase in estradiol [4,28] and, probably, progesterone levels, demonstrating the return of the action of the axis on the female reproductive system.

The limitations of this study include the inability to longitudinally follow BFM-donors from the immediate postpartum period to one year after delivery, although this was the original study design. Initiating recruitment within 15 days postpartum would have required enrolling a large number of women without any guarantee that they would breastfeed and subsequently become BFM-donors, resulting in an even greater challenge in obtaining an adequate number of eligible participants. This approach would also have increased the risk of sample attrition, costs, and timeline delays, particularly given the limited resources available and the restrictions imposed by the COVID-19 pandemic. Furthermore, during part of this period, vaccines were not yet available for breastfeeding mothers, which led to a high rate of refusal to participate in the study.

Additionally, as this is an observational study, causality cannot be established. Finally, the findings reflect the specific characteristics of the BFM-donors included in this study and, therefore, may not be generalizable to all breastfeeding mothers or HM-donors.

All the changes found suggest that further studies be carried out to deepen and elucidate the effects of BF on bone metabolism.

In conclusion, HM donation did not exacerbate lactation-related BMD reductions. Bone mineral density recovered substantially between 6 and 12 months postpartum. Comparing BFM-donors at 12 months, versus BMF-controls at 15 days, even with continued complementary breastfeeding, densitometric results tended to return to the means seen 15 days postpartum. These alterations were not influenced by the volume of HM donated, demonstrating that donation does not promote additional changes to bone mass beyond what is already expected, temporarily, by the practice of breastfeeding.

## Data availability statement

The data that support the findings of this study are available from the corresponding author.

## Conflicts of interest

The authors declare no conflicts of interest.

## Appendix Supplementary materials

Image, application 1
