# Supplementary material for: Does donating human milk result in greater compromise to the bone mass of breastfeeding mothers?
Source: J Pediatr (Rio J). 2026 Sep 5;102(6):101604. doi: 10.1016/j.jped.2026.101604 (PMC13571993; doi:10.1016/j.jped.2026.101604)
Supplement: Supplementary file 1 [file mmc1.docx]

**JPED-D-26-00168_Supplementary Material**

| **Supplementary Table 1** Spearman correlation coefficients and corresponding p-values (n = 39) for the associations between donated human milk volume and bone mineral density measurements and bone biomarkers. | |
| --- | --- |
|  | **Human milk donated r (p)** |
| Total body BMD (g/cm^2^) | -0.045 (0.781) |
| Total body BMC (g) | -0.175 (0.285) |
| Femur BMD (g/cm^2^) | -0.136 (0.407) |
| Femur BMC (g) | -0.287 (0.076) |
| Lumbar BMD (g/cm^2^) | -0.040 (0.808) |
| Lumbar BMC (g) | 0.017 (0.913) |
| BAP (mcg/L) | 0.273 (0.092) |
| OC (ng/mL) | 0.197 (0.228) |
| S-CTx (ng/mL) | 0.237 (0.145) |

| **Supplementary Table 2** Pearson's correlation coefficients and corresponding p-values. | | | |
| --- | --- | --- | --- |
|  | **BAP (mcg/L)**  **r (p)** | **OC (ng/mL)**  **r (p)** | **S-CTx (ng/mL)**  **r (p)** |
| Total body BMD (g/cm^2^) | 0.112 (0.494) | -0.133 (0.417) | -0.284 (0.007) |
| Total body BMC (g) | 0.191 (0.242) | -0.024 (0.882) | -0.214 (0.190) |
| Femur BMD (g/cm^2^) | 0.281 (0.082) | 0.179 (0.275) | -0.051 (0.756) |
| Femur BMC (g) | 0.233 (0.152) | 0.102 (0.533) | -0.060 (0.714) |
| Lumbar BMD (g/cm^2^) | 0.355 (0.054) | 0.096 (0.576) | -0.035 (0.182) |
| Lumbar BMC (g) | 0.310 (0.026*) | 0.092 (0.557) | -0.202 (0.836) |
